# Chemical and Biological Properties of *Hymenaea courbaril* L.: A Review

**DOI:** 10.1002/cbdv.202503154

**Published:** 2025-12-19

**Authors:** Cicera Alane Coelho Gonçalves, Geane Gabriele de Oliveira Souza, Ana Maria Fernandes Duarte, Joice Barbosa do Nascimento, Fázia Fernandes Galvão Rodrigues, José Galberto Martins da Costa, Fabíola Fernandes Galvão Rodrigues

**Affiliations:** ^1^ Department of Biological Chemistry Regional University of Cariri Crato Brazil; ^2^ Research Laboratory of Natural Products Department of Biological Chemistry Regional University of Cariri Crato Brazil

**Keywords:** bioactivities, ethnopharmacology, *Hymenaea courbaril* L., Jatobá, phytochemistry

## Abstract

*Hymenaea courbaril* L., commonly known as jatobá, is a plant traditionally used by native populations for medicinal purposes, in addition to providing edible fruits and durable wood for construction. In Brazilian traditional medicine, products from this species are employed to treat wounds, inflammations, bacterial infections, rheumatism, anemia, respiratory and gastric disorders, bronchitis, and prostate conditions. This review aimed to summarize the phytochemical composition and biological activities of *H. courbaril* L., establishing a foundation for future studies. The species is rich in bioactive compounds, including flavonoids, terpenes, phenols, and coumarins present in its leaves, fruits, bark, and resin. Scientifically validated bioactivities include antibacterial, antifungal, antiviral, anti‐inflammatory, antioxidant, muscle‐relaxant, and antiproliferative effects. Low acute toxicity has been demonstrated in in vivo assays. Despite these promising results, gaps remain regarding its chemical characterization, medicinal, and nutraceutical potential. This review highlights the need for further research to validate traditional uses, ensure safe applications, and explore new therapeutic prospects.

## Graphical abstract

PRISMA‐style flowchart showing the screening process of studies identified and selected in the review has been added to the attachments.

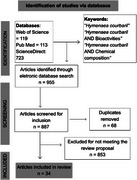

## 1 Introduction

The genus *Hymenaea* (Fabaceae) comprises 14 species widely distributed in tropical and subtropical regions [1]. It consists of trees that are exploited for various purposes. The plant species of greatest economic importance within the genus is *Hymenaea courbaril* L., popularly known as jatobá. This plant is used by native populations for medicinal purposes and in civil construction, and its fruits provide a nutritious food source for a variety of organisms [2, 3].

Species of *Hymenaea* spp. exhibit a neotropical distribution, occurring in eastern Africa as well as in central and south America. In Brazil, these plants are used in traditional medicine for the treatment of wounds, inflammations, bacterial infections, rheumatism, anemia, respiratory and gastric disorders, bronchitis, and prostate diseases [4]. Recent scientific studies have demonstrated the presence of terpenes, phenolic compounds, and other substances, which may be responsible for the efficacy in the treatment of the aforementioned ailments [5]. Furthermore, the bark, resin, and leaves of this plant have been used for the relief of conditions such as rheumatism, arthritis, and respiratory diseases [6].

The numerous bioactivities of extracts obtained from different parts of this species have been investigated and reported in the literature, with emphasis on antimicrobial activity against multidrug‐resistant bacteria of significant clinical interest, as well as its strong antioxidant activity [7]. Thus, given the therapeutic relevance and pharmacological potential of *H. courbaril* L., this work aims to conduct a bibliographic survey of its chemical constituents and bioactive properties, to contribute to future scientific investigations and therapeutic applications.

## 2 Methodology

Information regarding the chemical composition and bioactivities of *H. courbaril* was collected using the Web of Science, ScienceDirect, and PubMed databases. For this investigation, the following search terms were used: “*Hymenaea courbaril*,” “*Hymenaea courbaril* and bioactivities,” and “*Hymenaea courbaril* and chemical composition,” in Portuguese and English, all articles published between 1990 and 2025 were analyzed, with only those studies aligned with the objective of this research being included.

Initially, 119 articles were found on Web of Science, 113 in PubMed, and 723 in ScienceDirect, totaling 955 studies. All studies identified during the search were evaluated, and refinement of the selection was carried out using specific inclusion and exclusion criteria. The inclusion criteria were based on experimental articles that contained in their title, abstract, or keywords, information regarding the bioactive properties and chemical composition of extracts derived from parts of the plant species *H. courbaril* L. The excluded studies were characterized as follows: studies that did not fall into the category of original research (such as letters to the editor, prefaces, commentaries, editorials, reviews, case reports, review articles, books, book chapters, theses, and dissertations); incomplete or inconclusive studies; and duplicate studies.

After a thorough analysis of the data according to the criteria listed above, 34 articles were included in this research. From these selected studies, the following information was collected: the plant part used and the types of solvents employed for the production of extracts and fractions, as well as the techniques for identification and isolation of chemical compounds, in addition to the bioactivities associated with isolated compounds, extracts, and fractions from different parts of the plant species *H. courbaril* L.

## 3 Botanical Aspects

**FIGURE 1:**
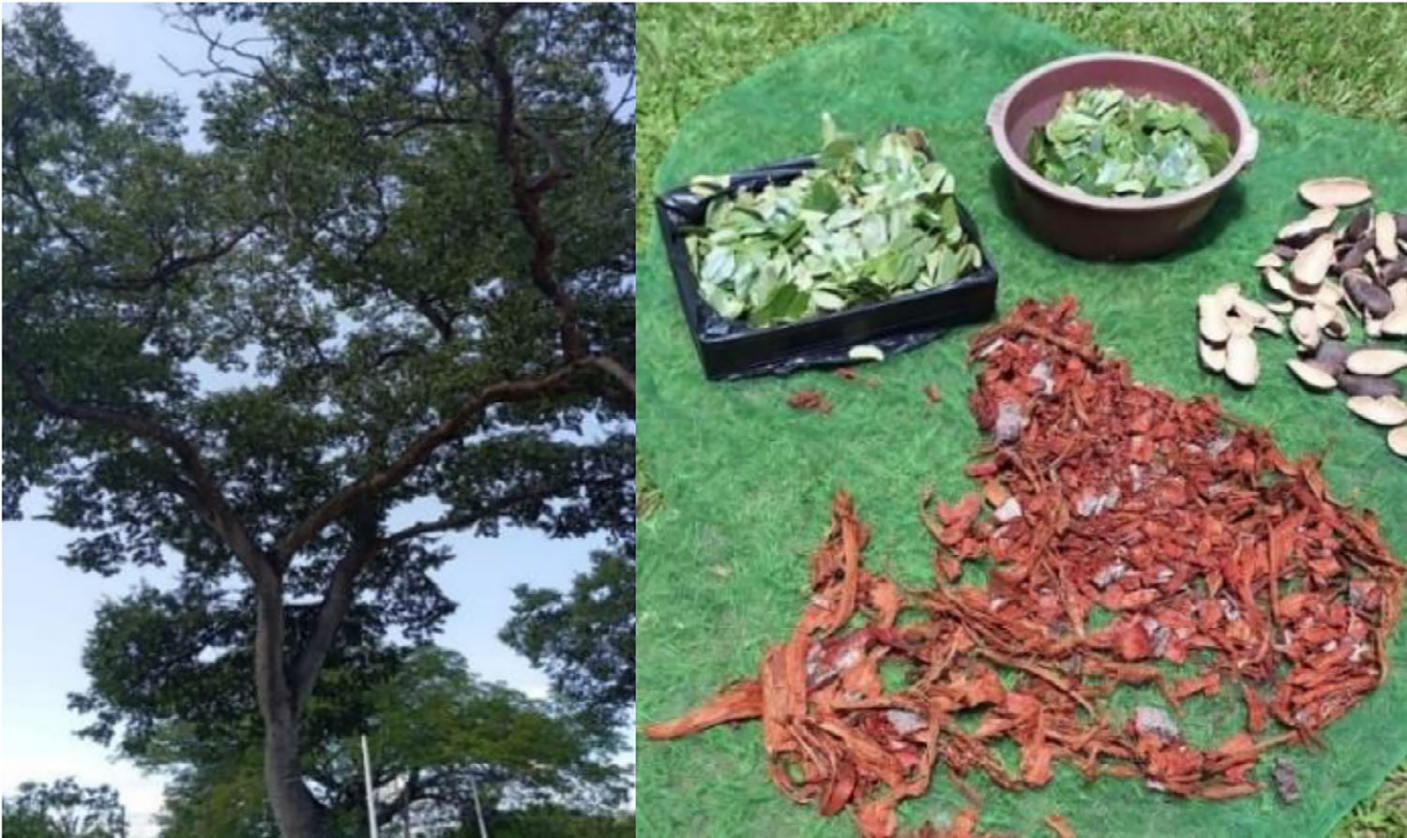
(A) *Hymenaea courbaril* L; (B) stem bark, leaves, and fruits of *H. courbaril* L.

*H. courbaril* L. is widely distributed in tropical forests of South America, with particular occurrence in the Atlantic Forest. It is characterized by compound leaves with two leaflets, inflorescences arranged in terminal panicles, and indehiscent pod‐like fruits. Each fruit contains six to eight seeds surrounded by an edible farinaceous pulp of high nutritional value, widely consumed by humans and various animals, especially rodents [8]. Jatobá is a large tree, reaching between 15 and 20 m in height, as illustrated in Figure 1. Its edible fruits have a high content of dietary fiber, calcium, and magnesium [9].

Morpho‐anatomical studies reveal leaves with well‐developed vascular bundles and epidermal cells adapted to environmental variations. The presence of resin secretory cavities in the stem indicates the production of secondary metabolites, such as terpenes and flavonoids, which confer ecological resistance and reinforce its therapeutic potential [4]. Most species of the genus have short paniculate inflorescences with white, robust flowers [10], occurring in a scattered manner in upland forests and certain high plains, most frequently in clayey and nutrient‐poor soils [11].

The fruit morphology differs from other species by exhibiting a thick rind and relatively heavy seeds. According to Da Silva and Lamarca [12], attributes such as pulp and bark yield are essential for understanding the reproductive biology and cultivation potential of the species. Seed germination rates are directly influenced by environmental factors, such as humidity and temperature. Seed dispersal is facilitated by interactions with large frugivorous animals, including birds and mammals, which contribute to the spatial distribution of natural populations [13].

From a morphological perspective, the species exhibits compound bifoliolate leaves and fruits with a thick endocarp, containing seeds surrounded by aromatic farinaceous pulp—features that are important for its identification and use in agroforestry systems [14]. In anatomical terms, structures such as resin secretory canals, paracytic stomata, and collateral vascular bundles stand out, demonstrating the structural complexity of the plant and its multiple applications [8]. Biometric studies indicate a wide intraspecific variability in fruits and seeds, which is crucial information for conservation strategies and genetic improvement programs [15].

Historically, traditional communities have recognized *H. courbaril* L. as a species of wide utility. Its dense and durable wood is highly valued for applications such as carpentry and civil construction. In parallel, parts such as the bark, resin, and pulp have been traditionally used in folk medicine, especially for the treatment of respiratory ailments and inflammatory conditions [12]. The significant presence of bioactive compounds, such as flavonoids and tannins, has attracted scientific interest due to their proven antioxidant, antimicrobial, and anti‐inflammatory properties [4]. Thus, *H. courbaril* L. emerges as a species of high ecological, economic, and therapeutic value. Its fruits play a crucial role in natural reproduction and dispersal, while the secondary metabolites present in the plant highlight a vast potential for exploration in the fields of phytotherapy, functional nutrition, and plant biotechnology.

## 4 Geographical Distribution and Ecology of the Species

The genus *Hymenaea* comprises approximately 14 predominantly Neotropical species, recognized both for their medicinal use and the exploitation of their robust timber. In Brazil, *H. courbaril* L. occurs across several biomes, including the Amazon, Atlantic Forest, Cerrado, Caatinga, and Pantanal, covering an extensive territorial range that encompasses nearly all states of the country. The species exhibits high ecological plasticity, occurring in diverse vegetation formations, from riparian and ombrophilous forests to restingas and upland forests, with an altitudinal distribution ranging from 0 to 1500 m [2, 16, 17].

Furthermore, jatobá demonstrates tolerance to adverse environmental conditions, such as prolonged droughts, mild frosts, and sloped soils. This species is able to survive periods of temporary or continuous flooding. Its phenology shows flowering between September and December, while fruiting occurs from June to December, with regional variations. Given its ecological importance, *H. courbaril* L. has been considered a priority species in genetic conservation efforts of forest trees. Its preservation is essential not only to maintain local biodiversity but also for the restoration of degraded ecosystems and the reinforcement of native flora [14, 18].

## 5 Traditional Use

*H. courbaril* L. is traditionally recommended by rural communities in the Northern region for the preparation of teas from its leaves, aimed at treating anemia and liver disorders. Other parts of the plant, such as the stem bark, inner bark, and seeds, are used in the preparation of teas and poultices for the treatment of respiratory and digestive system diseases, as well as ailments related to the blood and hematopoietic organs [19, 20].

Accordingly, ethnobotanical studies have been conducted to document the traditional uses carried out by various communities. To this end, Lago et al. [20], they documented that riverine communities of the Unini River in Amazonas use the plant's resin for the treatment of flu, sore throat, and cough. de Nazaré da Silva Braga et al. [21], in their study, they also identified that communities in the same region use other parts of jatobá, including bark and seeds, to prepare teas for the treatment of hepatitis, kidney diseases, high blood pressure, diabetes, and anemia. In the municipality of Anapu, in Pará, they commonly use, in addition to the bark and resin, the flowers in tea form, both used for the treatment of anemia, flu, cough, bronchitis, inflammation, wound healing, intestinal pain, and as an anthelmintic [22]. In another community located in northern Brazil, the bark and sap are commonly used in the preparation of teas and oral syrups, treating issues such as indigestion, inflammation, ulcers, and respiratory conditions, including flu, bronchitis, and throat problems [23].

Jatobá is also used by farmers in three municipalities in the northern region of the state of Mato Grosso in 42 different ways, with a total of 120 citations classified into six categories (food, handicraft, ecological, timber, medicinal, and technological), with the medicinal category being the most frequently mentioned by respondents (79%; *N* = 95) [24]. These findings suggest that the local population recognizes and values the medicinal properties of the species, which may be related both to traditional knowledge of its numerous benefits and to the lack of or limited access to conventional healthcare services. Complementarily, 25 individuals residing in a municipality in the state of São Paulo, in southeastern Brazil, use decoctions or syrup made from jatobá to treat flu [25]. Traditional use reports of the species also include the treatment of bronchitis, pneumonia, various inflammations, and flu‐like symptoms, conditions for which the activity of bioactive compounds such as terpenes and phenolics is scientifically recognized [5].

In addition, in the Chapada do Araripe, located in the state of Ceará, traditional communities use the inner bark and the fruit to treat the aforementioned ailments, as well as fever, cough, and to “thin the blood” (a popular expression often associated with improved circulation). It was also observed that the preference for traditional medicine among local inhabitants is largely due to its low cost, as well as being an integral part of their traditions and social interactions. Furthermore, its use is also widespread among Quilombola communities and by numerous other communities across several Brazilian states [26, 27, 28].

In light of the above, the significant medicinal value attributed to the species stands out, highlighting its phytotherapeutic potential to be explored in future pharmacological studies, especially if supported by consistent and recurrent reports of efficacy. It is important to emphasize that this type of empirical knowledge, transmitted across generations, constitutes an important source of information for ethnobotanical research and can serve as a basis for more in‐depth scientific investigations. That said, the importance of using products derived from this species is directly related to its conservation, as it is widely exploited for medicinal and nutraceutical purposes. Therefore, literature reviews provide a foundation to support experimental research that can scientifically validate such uses, enabling the safe consumption of products derived from jatobá, while also valuing the traditional knowledge of communities that is passed down from generation to generation.

## 6 Nutraceutical Value

Beyond its medicinal value, the recognition of the plant's nutraceutical properties is highly relevant, as it highlights its potential not only as a therapeutic agent but also as a functional food capable of promoting health benefits and preventing diseases. These applications enhance its value, justifying interest in its inclusion in food formulations, supplements, and wellness products. Accordingly, the fibrous residue and pulp flour of jatobá have been evaluated for Santos et al. [28]. showing high protein content (11 and 12 g/100 g) and dietary fiber (49 and 44 g/100 g). The fibrous residue exhibited the highest total fiber content, as well as vitamin C. The pulp flour and the sap presented 462 mg GAE/100 g and 181 mg GAE/100 g, respectively.

In this context, Almeida et al. [29]. also identified the presence of carbohydrates, lipids, and proteins in the fruit pulp, as well as carotenoids, phenolic compounds, and vitamin C. Corroborating this, Maria de Los et al. [30] investigated the hydrocolloids extracted from the pulp and seeds of jatobá, highlighting their high content of carbohydrates, fibers, and minerals, as well as good emulsifying properties. The results indicate that these compounds are promising for use as functional ingredients in foods and edible films. These bioactive components not only provide basic nutritional support but also act as natural antioxidants [31], improving inflammatory processes, combating oxidative stress, and promoting overall health. Therefore, its chemical characterization further increases interest in the use of products derived from *H. courbaril* L. both in nutrition and in the development of nutraceutical products.

Despite the absence of direct evidence in the literature supporting some specific applications, its use by the population can be partially justified, given the various biological activities attributed to this plant in previously published studies, activities that are directly linked to the presence of the chemical compounds already mentioned. Accordingly, ethnobotanical studies have been conducted to document the traditional uses carried out by various communities.

## 7 Chemical Composition of *H. courbaril* L

The analysis of the chemical constituents of *H. courbaril* L. is essential to explore its biological potential, as this plant harbors a variety of bioactive compounds that can be applied in fields such as pharmacology, food health, and biotechnology. Despite its traditional use, *H. courbaril* L. remains poorly explored in terms of characterization, identification, and isolation of its bioactive compounds, as well as their application in biotechnological and pharmacological fields, as shown in Table S1.

The phytochemical characterization of secondary metabolites in this plant species is essential for the identification of biological markers, contributing to the elucidation of the main classes of compounds with bioactive potential. Among the identified constituents, flavonoids were predominant, representing the major fraction of the detected metabolites. Flavonoids are widely known for their numerous biological properties, as presented in Table S1. In addition, tannins, terpenes, coumarins, sterols, and carotenoids were also identified [31, 32, 33, 34, 35].

Table S1 shows that the techniques used for the identification and isolation of the chemical components of this species include ultrahigh‐performance liquid chromatography (UHPLC); gas chromatography (GC); mass spectrometry (MS); ultraviolet–visible (UV‐Vis) spectroscopy; high‐performance liquid chromatography (HPLC); diode array detection (DAD); and NMR—^1^H and ^13^C nuclear magnetic resonance spectroscopy. The chemical structures of the identified compounds are described in Figure 2.

FIGURE 2Chemical structures of the identified compounds in *Hymenaea courbaril* L.

**Figure:**
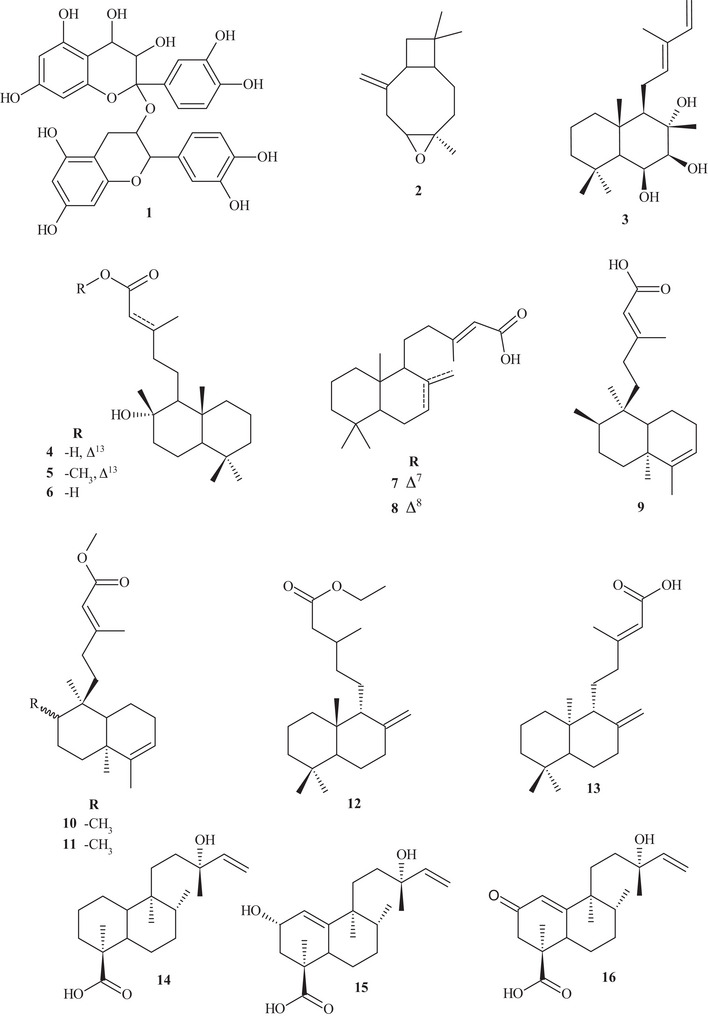

**Figure:**
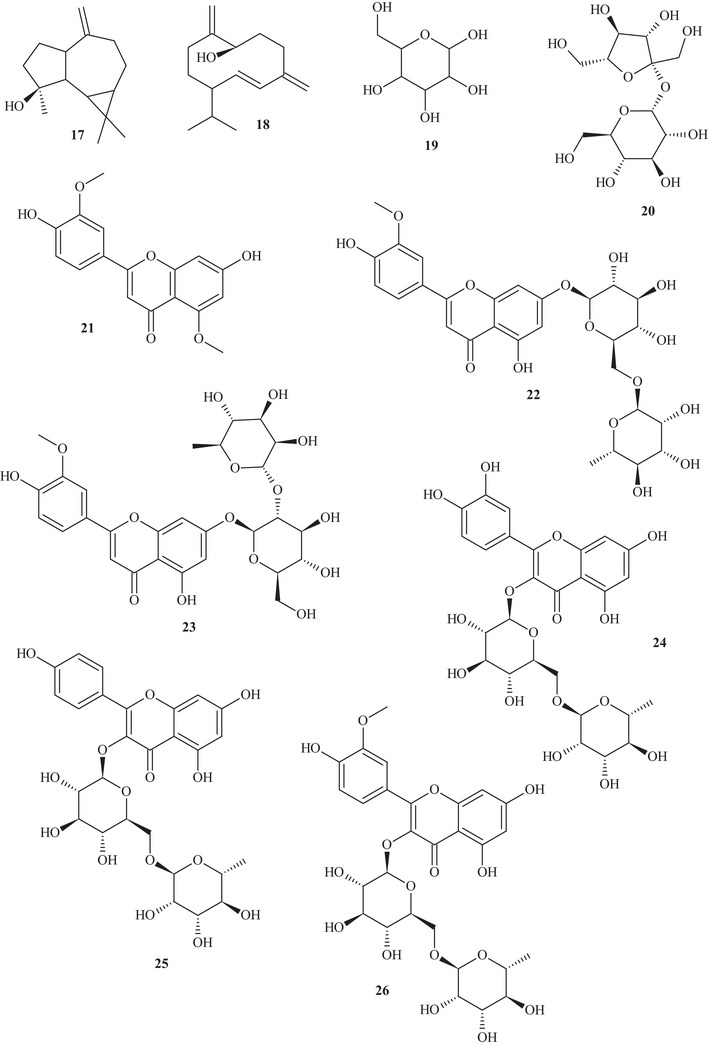

**Figure:**
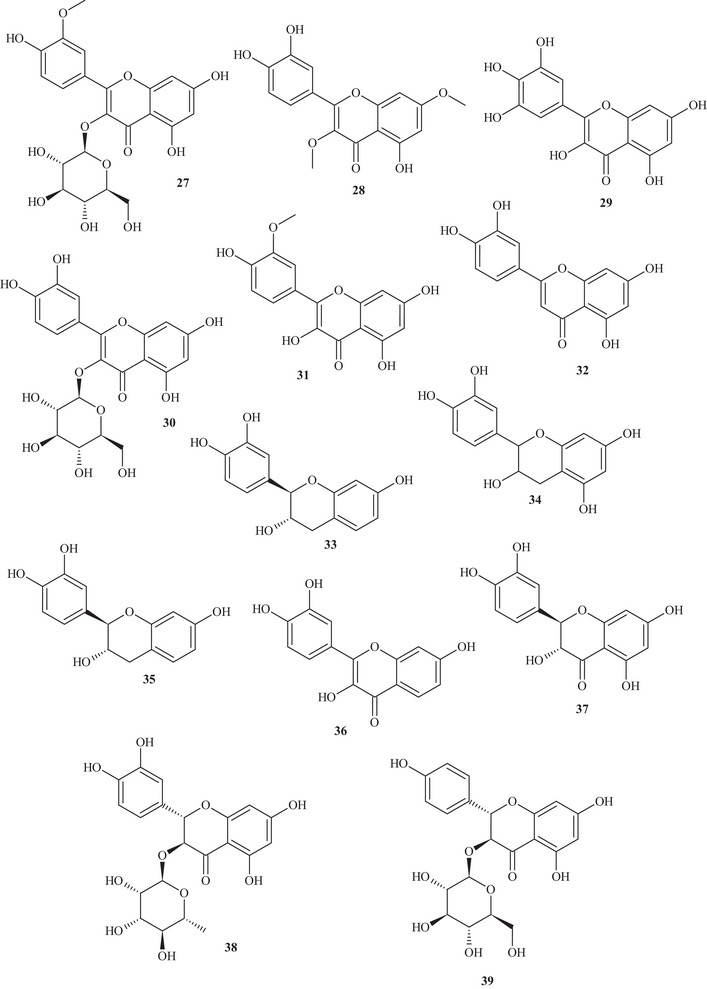

**Figure:**
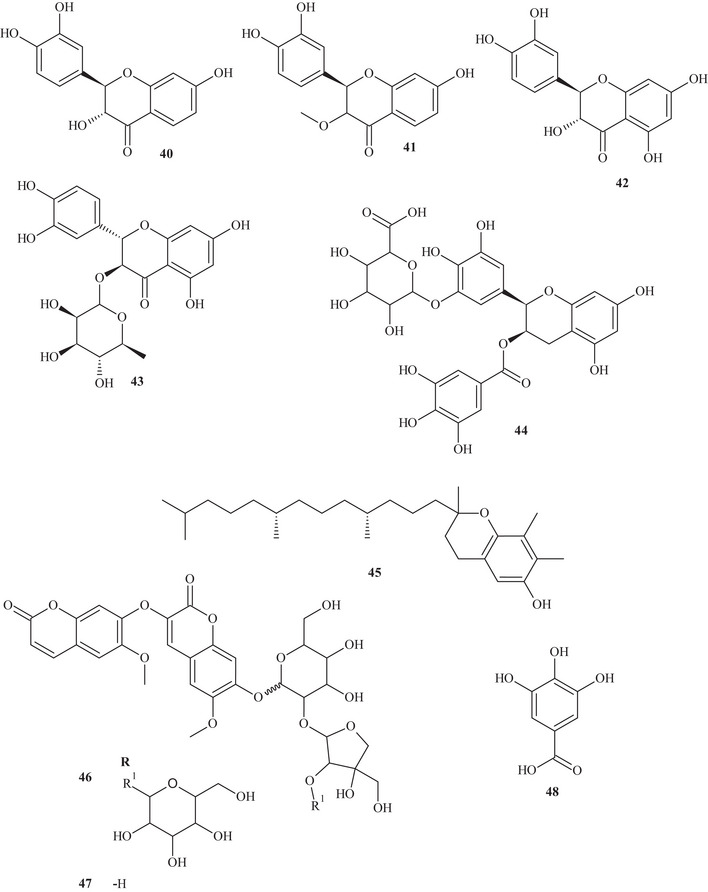

**Figure:**
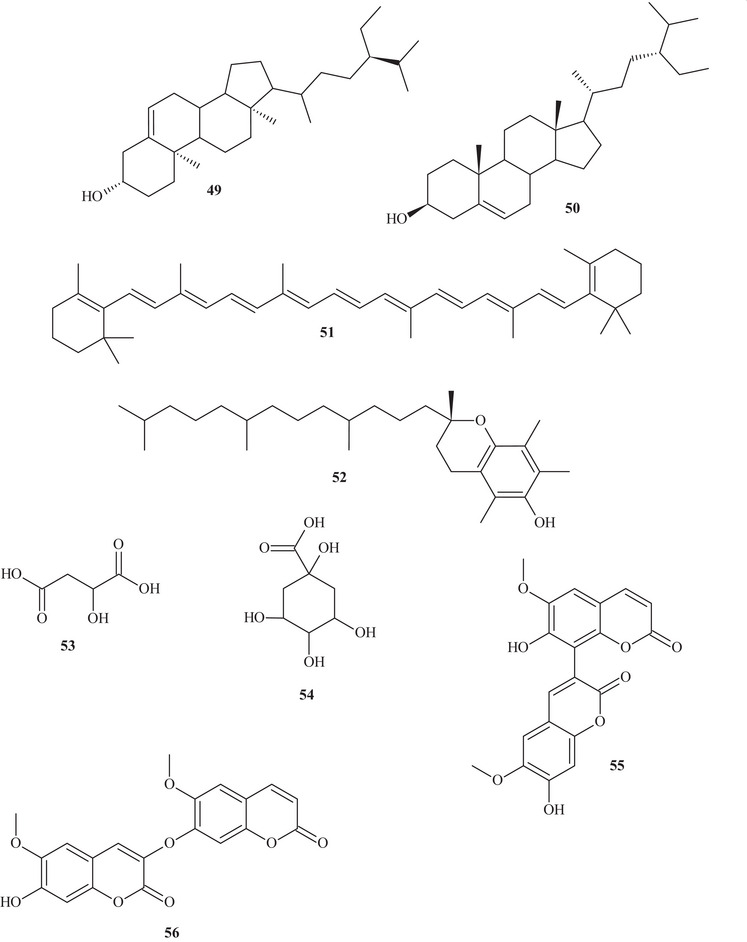

It is possible to observe that the most commonly used solvents for the extraction and isolation of compounds were polar, such as ethanol, ethyl acetate, and methanol. Solvent polarity plays a crucial role in the extraction of bioactive compounds, and evidence from the reviewed literature indicates that polar solvents are more efficient in extracting antioxidants from this plant [3, 34, 36, 37]. Thus, Santos et al. [35]. evaluated trunk extracts of *H. courbaril* prepared using the solvents cyclohexane, ethyl acetate, and methanol, identifying the presence of phenols, flavonoids, chalcones, alkaloids, catechins, and triterpenoids.

Cruz et al. [38] through UPLC–HRMS/MS chemical analysis, detected a high total phenolic content was observed, especially in the stem bark extract, and the plant was found to be rich in compounds with antioxidant activity. The study also showed that the leaves yielded a high content of polyphenols. It is possible to observe that phytochemicals extracted using polar media tend to exhibit higher pharmacological relevance due to their high reducing capacity and effectiveness in scavenging free radicals. The characterization and isolation of these compounds are fundamental to understanding their properties and potential applications. Research in this field may reveal characteristics such as antigenotoxic activity, antimicrobial activity as well as antibacterial activity against various multidrug‐resistant pathogens [39, 40]. Flavonoids comprise a group of polyphenolic compounds that are ubiquitous in plant species [40, 41]. Within this context, Almeida et al. [29]. evaluated the fruit pulps of *H. courbaril* L., observing the presence of high levels of ascorbic acid (47.5 mg/100 g).

Several bioactive compounds have been isolated from various parts of this plant, including the leaves, which exhibited large amounts of flavanone metabolic groups, followed by phenols and catechins. In contrast, a greater number of metabolite groups were observed in the bark, including flavonols, flavanones, flavanonols, xanthones, and leucoanthocyanidins [41, 42]. The phytoconstituents of the hydroalcoholic extract of the stem bark included coumarins, flavonoids, phenolics, tannins, and saponins, and HPLC analysis revealed that the flavonoid astilbin was the main component [40].

In addition, this plant species also produces a resin rich in terpenoid compounds, mainly diterpenes. The resin and other plant parts are used in traditional medicine for the treatment of various pathologies hey evidenced the presence of terpenes, flavonoids, and coumarins in the total ethanolic extract of the resin. These data are extremely important for understanding the bioactive properties, as well as the optimal extraction methods to achieve the highest yield of the plant's components. Despite these advances, it is evident that the number of studies remains very limited, given the medicinal importance of *H. courbaril* L., highlighting the need for further research to better understand its chemical nature and explore its therapeutic potential [34, 43].

## 8 Biological Activities of *H. courbaril* L.

### 8.1 Myorelaxant and Anti‐Inflammatory Activity

In the study of Bezerra et al. [33] the ethyl acetate fraction from the stem bark of *H. courbaril* L. showed a predominance of flavonoids. Within this fraction, the flavonoid astilbin was identified, which, when tested individually, demonstrated airway smooth muscle relaxation in rats, achieving 49.8% relaxation at a concentration of 1000 µg/mL. The fraction itself exhibited a similar effect, with 46.98% relaxation at 300 µg/mL, suggesting that astilbin may be directly responsible for the overall activity observed. Furthermore, the study demonstrated a reduction in bronchial hyperresponsiveness, along with a decrease in the total number of leukocytes, particularly eosinophils and neutrophils, in the bronchoalveolar lavage. This finding, not previously reported for the species, indicates that, under in vivo conditions, the fraction holds potential as both an anti‐inflammatory.

The bronchodilator and anti‐inflammatory effects observed may be mainly attributed to astilbin, which exhibited an activity level comparable to that of the whole fraction. In addition, the study reported a reduction in hyperresponsiveness, as well as a decrease in the total number of white blood cells, particularly eosinophils and neutrophils, in the bronchoalveolar lavage, suggesting that, under in vivo conditions, this fraction has the potential to act as an anti‐inflammatory agent.

The terpenes 4, 7, and 8 were evaluated for their anti‐inflammatory activity using cyclooxygenase‐1 (COX‐1) and cyclooxygenase‐2 (COX‐2) enzymes. The tested compounds demonstrated selective inhibitory activity against COX‐2, a highly desirable selectivity profile, since specific inhibition of COX‐2 is associated with the reduction of inflammatory and pain processes [6].

These findings suggest that the tested compounds have therapeutic potential as selective anti‐inflammatory agents. Further studies are warranted, particularly assays evaluating the blockade of inflammatory cytokines such as TNF‐α, IL‐1, and IL‐6. Although the activity of astilbin has been highlighted, other substances may also contribute synergistically to the observed effects, which justifies the need for future studies with other isolated compounds. Furthermore, more in‐depth investigations are required to elucidate the mechanisms of action of the compounds present in *H. courbaril* L., thereby supporting the development of phytotherapeutics or new anti‐inflammatory drugs with improved safety and efficacy profiles.

### 8.2 Antimicrobial Activity

The search for antimicrobial agents of natural origin is crucial in view of pathogen resistance, which threatens the effectiveness of many drugs. Within this context, compounds derived from natural sources, such as plants, may offer new mechanisms of action. Moreover, these agents may exhibit lower toxicity and consequently fewer side effects. Despite the challenges of extraction and purification, continuous research is essential to expand the therapeutic arsenal and effectively combat infections [44].

In this search for natural compounds with antimicrobial properties, Ribeiro et al. [45]. evaluated the efficacy of the ethanolic and hexanic leaf extracts of *H. courbaril* L. against *Leishmania amazonensis* was evaluated, showing notable leishmanicidal activity with IC_50_ values of 44.10 and 35.84 µg/mL, respectively. In addition, the ethanolic extract of *H. stignocarpa*, a species belonging to the same genus as the one addressed in this study, presented an IC_50_ of 4.69 µg/mL. These findings indicate that this genus of plants holds considerable antimicrobial potential to be further explored.

In addition, Almeida et al. [36] developed biofilms with concentrations of the ethanolic extract from the fruit bark, which demonstrated efficacy in inhibiting nine bacterial strains of significant clinical relevance, notably *Escherichia coli* (95.0% inhibition), *Bacillus cereus* (92.5%), *Bacillus subtilis* (96.5%), *Pseudomonas aeruginosa* (94.4%), *Salmonella typhimurium* (90.4%), and *Salmonella enteritidis* (93.9%). Similarly, Cruz et al. [38] investigated the inhibitory activity of biofilms against the Gram‐positive pathogen *Staphylococcus aureus* (ATCC 25923) using the ethanolic leaf extract of *H. courbaril* L., which stood out in comparison to the broad‐spectrum antibiotic chloramphenicol, showing inhibition rates of 78.29% and 78.85%, respectively.

They also evaluated the antibacterial activity of the ethanolic stem bark extract of jatobá against the Gram‐positive species *S. aureus* (ATCC 25923). The results showed that the extract exhibited a minimum inhibitory concentration (MIC) of 12.5 mg/mL [38]. Regarding antifungal activity, Costa et al. [42]. assessed the activity of the xylem sap of *H. courbaril* L., which inhibited the growth of dermatophytes and *Cryptococcus neoformans* with a MIC < 256 µg/mL, while compound fisetin showed an MIC < 128 µg/mL against these fungi.

For antiviral activity, ethanolic leaf extracts of *H. courbaril* L. exhibited strong in vitro activity against rotavirus. The extracts prevented the formation of cytopathic effects (CPE), and RT‐PCR analysis did not detect amplification of rotavirus genetic material. This activity is associated with the bioactive compounds detected in the extract, including tannins, flavonoids, saponins, coumarins, and terpenes, which were the main classes of natural compounds identified [33].

The crude extract and protein fractions from the seeds of *H. courbaril* L. were investigated for the presence of inhibitors of the proteolytic enzymes trypsin and papain, as well as for antimicrobial activity against *Vibrio parahaemolyticus*, *S. aureus*, and *E. coli*. The protein fractions were obtained from the crude extract after ammonium sulfate precipitation at three saturation ranges (0%–30%, 30%–60%, and 60%–90%), referred to as Hc030, Hc3060, and Hc6090, respectively. Both the crude extract and the protein fractions inhibited enzyme activity, although to varying degrees. Antimicrobial activity was observed in fractions Hc030 and Hc3060, but only against *V. parahaemolyticus* [46].

Furthermore, the seed extracts prepared with 70% and 80% ethanol inhibited most bacterial strains, particularly *P. aeruginosa*, showing the lowest MIC values (1.0–8.0 µg/mL), compared to the bark extracts. The combination of 70% ethanol extracts from the bark and seeds reduced the inhibitory concentration by approximately 4‐ to 32‐fold against *B. cereus*, *B. subtilis*, *Enterococcus faecalis*, *S. aureus*, *P. aeruginosa*, and *S. enteritidis*, compared to each extract alone. These findings suggest that natural extracts of *H. courbaril* L. exhibit antibiofilm, antimicrobial, antibacterial, antiviral, and antifungal properties, emerging as promising alternatives against infections associated with microorganisms resistant to conventional antibiotics [7].

Although promising, the available results are preliminary and present significant gaps that limit their application and pharmaceutical development. Many studies have been conducted only in vitro, without validation in in vivo models. Furthermore, most research has focused on extracts obtained from different parts of the species, with scarce data on isolated compounds and the molecular mechanisms involved.

Therefore, to overcome these limitations, it is essential to conduct preclinical and clinical studies to evaluate the efficacy of the extracts or isolated compounds in animal models. Mugali et al. [43]. also employing integrative approaches, such as molecular biology, metabolomics, and computational modeling, to elucidate the mechanisms of action and provide advances in the therapeutic development of the species.

### 8.3 Antioxidant Activity

Natural products with antioxidant potential play a crucial role in neutralizing free radicals, protecting cells against oxidative damage. Several studies indicate that compounds present in plant extracts possess significant antioxidant properties, contributing to the prevention of chronic diseases and promoting overall health. Thus, Carneiro Lobo et al. [40] performed the assay using the 1,1‐diphenyl‐2‐picrylhydrazyl (DPPH•) radical, demonstrating that the hydroalcoholic extract of the stem bark of *H. courbaril* exhibited strong antioxidant activity, with an IC_50_ of 3.12 µg/mL.

Veggi et al. [47] extracted polyphenolic compounds from the bark of *H. courbaril* L. using CO_2_ and water, and this extract exhibited high antioxidant activity (IC_50_ of 0.2 mg/cm^3^). This behavior can be explained by the antioxidant properties of proanthocyanidins (tannins), which are potent natural phenolic compounds in scavenging free radicals. In addition, the powder from the dried bark also contained a high amount of procyanidins (condensed tannins), which exhibit a variety of biological activities, including strong antioxidant activity [48].

Several studies have shown that natural extracts exhibit significant antioxidant activity, being comparable to, or in some cases even superior to, α‐tocopherol, a widely recognized antioxidant for its effectiveness in neutralizing free radicals. Suzuki et al. [49] evidenced that the methanolic extract of the heartwood of *H. courbaril* L. exhibited the highest level of activity (EC_50_ = 44 mg/L), surpassing that of α‐tocopherol (EC_50_ = 48 mg/L) in the DPPH• (2,2‐diphenyl‐1‐picrylhydrazyl) assay. These findings reinforce the potential of such natural compounds in mitigating oxidative stress and protecting against cellular damage.

In this context, Suzuki et al. [49]. and Imai et al. [32] identified that the IC_50_ values in the DPPH• assay (2,2‐diphenyl‐1‐picrylhydrazyl) for compounds 32 and 33, isolated from the heartwood extract, were 28 and 48 µg/mL, respectively, both more efficient than α‐tocopherol (IC_50_ = 51 µg/mL). Concomitant with these promising results, Cruz et al. [38] in the antioxidant ferric reducing antioxidant power (FRAP) assay, it was observed that the lyophilized ethanolic extract of the leaves exhibited the highest Fe^3+^ reducing potential, with a value of 834.3 mg BHTE/g, followed by the seed extract (802.0 mg BHTE/g) (*p* < 0.05).

The antioxidant potential of hydroalcoholic extracts from the bark and seeds of *H. courbaril* L. was evaluated, with the seed extract obtained using 80% ethanol exhibiting the highest antioxidant potential in the ABTS^+^• (2,2′‐azino‐bis(3‐ethylbenzothiazoline‐6‐sulfonic acid), DPPH• (2,2‐diphenyl‐1‐picrylhydrazyl), and FRAP assays. This extract also showed the highest total phenolic content (5135.61 GAE/100 g dry residue), surpassing the maximum value obtained for the bark extract with 50% ethanol (2614.74 GAE/100 g dry residue) [7]. In the same context, the pulp of *H. courbaril* was also evaluated, showing a value of 5.2 mM/g in the ABTS^+^• assay, standing out as one of the tested species with the highest antioxidant activity [29].

These evidences highlight the importance of continued investigation into the use of natural antioxidants in the prevention of diseases associated with redox imbalance, and reinforce the potential of compounds derived from *H. courbaril* L. as promising candidates for pharmaceutical and nutraceutical applications in the development of new therapeutic strategies derived from *H. courbaril* products, despite the excellent antioxidant potential demonstrated, further investigations are still required to address existing knowledge gaps and consolidate their therapeutic applicability.

In this context, in the study conducted by Santos et al. [28] by‐products derived from *H. courbaril* L. were tested for their potential antioxidant effects, with the fibrous pulp residue showing the highest antioxidant capacity in the DPPH, FRAP, and ABTS assays. This result can be explained by the presence of compounds such as vitamin C and total phenolic content. It is noteworthy that most of the studies mentioned evaluated the products using assays based on free radical scavenging capacity. However, complementary methods, such as the deoxyribose protection assay and the linoleic acid/β‐carotene co‐oxidation assay, can provide a more comprehensive evaluation of antioxidant potential, allowing more precise inferences regarding the different mechanisms of action involved.

### 8.4 Toxicological and Cytotoxicity Evaluation

Given the bioactivities listed above, it is essential to assess the toxicity of the various parts of this plant to ensure the safety of its therapeutic or traditional use. Accordingly, the literature reports several important tests aimed at clarifying the potential adverse effects of this species. One of the tests conducted was the micronucleus assay using mouse bone marrow, which revealed that the sap extracted from the trunk of *H. courbaril* L. did not exhibit cytotoxic, clastogenic, or aneugenic effects, nor did it show anticytotoxic, anticlastogenic, or antianeugenic activity. Similarly, the leaf extract did not display cytotoxicity at 50 µg/mL and was considered toxic only at high concentrations, such as 5000 µg/mL [37, 47].

These results suggest that, although there is no evidence of significant adverse effects at the concentrations analyzed, the sap and leaf extract did not exhibit protective properties against cellular or genotoxic damage. In contrast, the hydroethanolic extract of the seeds demonstrated antigenotoxic effects and cytotoxic activity against B16F10 melanoma cell lines, showing both dose‐ and time‐dependent responses [37].

In addition, the jatobá resin was analyzed in vivo for its toxic and genotoxic effects using the model organism *Drosophila melanogaster*. Different concentrations of the extracts were tested, with larvae showing greater susceptibility to the partial ethanolic extract. Somatic mutation and mitotic recombination were assessed in the eyes of the flies, and significant genotoxic alterations were observed when exposed to the partial extract, suggesting that the absence of coumarins quantified in this extract may increase the insect's sensitivity to genetic material damage [41].

To evaluate the toxicological profile of the stem bark, the Ames mutagenicity assay was employed. The hydroalcoholic extract exhibited low mutagenic potential without metabolic activation, with effects observed only at the highest concentrations. In addition, the extract affected the survival of the nematode *Caenorhabditis elegans* only at concentrations of 800 and 1600 µL/mL, suggesting a relatively wide safety margin for use at moderate doses [40]. The isolated compound fisetin showed lower toxicity (IC_50_ = 158 µg/mL) compared to the fresh xylem sap (IC_50_ = 109 µg/mL), suggesting safety for the use of this natural compound, although further studies are necessary to ensure and confirm its therapeutic potential [42].

These data indicate that the different extracts of *H. courbaril* generally exhibit low toxicity, as evidenced by in vitro and mutagenicity assays. However, further studies are required in this regard, as there are numerous gaps concerning the toxicological profile of the plant's parts. Therefore, chronic toxicity and carcinogenicity assays are necessary to evaluate the effects of prolonged use.

## 9 Biotechnological Potential and Associated Patents

The interest in the various bioactive properties of *H. courbaril* L. is also reflected in the filing of patents involving the species, covering phytotherapeutic, cosmetic, and food formulations. Accordingly, patent records were found in INPI and on the WIPO website involving derivatives of this plant species, published between 2012 and 2023.

Application number:

**PI 1000862‐4 A2** (January 17, 2012): The patent titled “Tissue adhesive produced from Brazilian flora species (*Brosimum parinarioides*, *Protium heptaphyllum*, and *H. courbaril* L) metabolized by the bacterium *Caulobacter crescentus* and preparation method,” registered in Brazil, describes a tissue adhesive applied in medicine and dentistry. This material exhibits bioadhesive, antimicrobial, and anti‐inflammatory properties, capable of promoting effective wound healing without the need for sutures, representing an innovative, sustainable alternative free of animal‐derived inputs, unlike traditional tissue adhesives.

**102023001040** (January 19, 2023): This invention concerns an antioxidant, photoprotective, and thermo‐responsive nanoemulgel based on *H. courbaril* bark extract. Its objective is the formulation and use of a nanoemulgel for the cosmetic sector with antioxidant and photoprotective properties, containing ethanolic extract of *H. courbaril* bark, *Linum usitatissimum* seed oil, ethylene oxide, and propylene oxide. The proposal emphasizes the value of Brazilian biodiversity and the application of innovative technologies, although its efficacy and safety require validation through comprehensive dermatological and physicochemical testing.

**BR 10 2012 017045 0 A2** (February 18, 2014): Medicinal composition of chicken egg and jatobá sap (*H. courbaril*). This invention patent concerns a medicinal composition combining chicken egg—served hot, cooked, or warm—with the pulp of the jatobá fruit (*H. courbaril*), resulting in a formulation intended for the treatment and relief of pain commonly referred to as “bicos de papagaio” (vertebral osteophytes). From an ethnopharmacological perspective, the approach is notable. However, there is a lack of robust scientific evidence supporting the efficacy and safety of this composition, particularly regarding potential interactions between its components. Clinical studies would be essential to validate this therapeutic application.

**BR 10 2013 033232 1 A2** (September 22, 2015): Broths using plant hemicellulose. This invention relates to the development of edible liquids enriched with xyloglucan extracted from the endosperm of the legume *H. courbaril* L., serving as a source of soluble fiber to improve nutritional quality, while providing desirable chemical and physical properties. The proposal is promising from both a nutritional and technological perspective; however, the functional efficacy and stability of the formulation still require detailed studies to ensure its safe and effective use in food products. (FUNDAÇÃO EDSON QUEIROZ, BR/CE).

**PI 0914137‐5 B1** (October 20, 2015): Process for obtaining polysaccharides from *H. courbaril* L. seeds and a cosmetic composition comprising these polysaccharides (Natura Cosméticos S.A., BR/SP). This proposal for obtaining polysaccharides for use in cosmetic formulations, as presented by Natura Cosméticos, highlights the potential of Brazilian biodiversity in biotechnological innovation. The polysaccharides, known for their moisturizing properties, may provide functional benefits in dermocosmetic formulations. However, the efficacy of these compounds must be validated through physicochemical and clinical studies to confirm their performance.

**BR 11 2019 009838 0 A2** (August 20, 2019): Anti‐cellular senescence cosmetic composition, use of *H. courbaril* L. derivatives, method for preventing cellular senescence, and method for modulating beta‐galactosidase expression. The use of plant extracts with potential to delay cellular processes associated with skin aging is promising, but requires validation through molecular and clinical studies to confirm the efficacy and safety of the active ingredient, as well as its actual capacity to influence specific biological pathways involved in senescence (Natura cosméticos S.A. BR/SP).

**102017025897** (December 1, 2017): This invention describes the production of xyloglucan microparticles extracted From *H. courbaril* L. seeds, used as encapsulating agents for ascorbic acid Through a spray‐drying process. This low‐cost technology produces high‐quality microparticles, applied in hamburger formulations, aiming to protect ascorbic acid from oxidation, preserve the sensory characteristics of the product during storage, and reduce the formation of undesirable compounds during cooking.

## 10 Conclusion

*H. courbaril* L. is a species of high relevance for local populations, particularly in the Brazilian semiarid region, due to its applications in traditional medicine. This plant is used in the treatment of various ailments and is also exploited for its considerable timber and nutraceutical potential, as well as for its antioxidant, anti‐inflammatory, antimicrobial, and cytotoxic activities. Despite its widespread use by communities across different regions, there is a scarcity of scientific studies that thoroughly evaluate its pharmacological, nutritional, and toxicological properties.

Therefore, there is a need for in‐depth scientific studies aimed at characterizing the chemical composition of the species and elucidating its biological properties, including the application of approaches such as metabolomics, molecular biology, and in silico modeling, which are emerging trends in medicinal plant research. Long‐term toxicological assays are also recommended.

The identification, isolation, and characterization of bioactive compounds responsible for the effects attributed to *H. courbaril* L. are essential steps for understanding its therapeutic potential. Such investigations are crucial not only to validate the traditionally attributed therapeutic efficacy of the species and ensure its safe use, but also to uncover its potential bioactivities and foster the development of new drugs with therapeutic applications.

## Author Contributions

**Cicera Alane Coelho Gonçalves**: conception and preparation of the original project, as well as data analysis and initial drafting of the manuscript, the critical review of the content was carried out, manuscript editing, references editing. **Geane Gabriele de Oliveira Souza**: Structure design. **Ana Maria Fernandes Duarte**: references editing. **Joice Barbosa do Nascimento**: The critical review of the content was carried out. **Fázia Fernandes Galvão Rodrigues**: The critical review of the content was carried out. **José Galberto Martins da Costa**: Supervision and writing. **Fabíola Fernandes Galvão Rodrigues**: manuscript editing, supervision and writing.

## Conflicts of Interest

The authors declare no conflicts of interest.

## Supporting information



The authors have cited additional references within the Supporting Information [50, 51, 52, 53, 54].
**Supporting File 1**: cbdv70789‐sup‐0001‐SuppMat.docx

## Data Availability

The data that support the findings of this study are available from the corresponding author upon reasonable request.
